# Gap-free telomere-to-telomere genome assembly of marbled flounder (*Pseudopleuronectes yokohamae*)

**DOI:** 10.1038/s41597-025-05942-5

**Published:** 2025-10-17

**Authors:** Weiwei Zheng, Aijun Cui, Zhen Meng, Ning Zhang, Yan Jiang, Jichang Zheng, Rixiang Zhao, Wenteng Xu, Yongjiang Xu

**Affiliations:** 1 State Key Laboratory of Mariculture Biobreeding and Sustainable Goods, Yellow Sea Fisheries Research Institute, Chinese Academy of Fishery Sciences, Qingdao, Shandong 266071 China; 2 Laboratory for Marine Fisheries Science and Food Production Processes, Qingdao Marine Science and Technology Center, Qingdao, Shandong 266237 China; 3 Yantai Zongzhe Marine Science & Technology Co., Ltd., Yantai, Shandong 265617 China

**Keywords:** Genomics, Sequencing

## Abstract

Marbled flounder (*Pseudopleuronectes yokohamae*) is becoming a commercially important flatfish species in Northeast Asia due to its strong environmental adaptability and excellent nutritional value. However, no high-quality marbled flounder reference genome was reported to date, which greatly limits the studies of evolutionary and functional genomics. Here, we reported the first gap-free T2T genome in flatfish (marbled flounder), with length of 582.73 Mb (contig N50: 26.29 Mb) combing short reads, PacBio HiFi long reads, ONT ultra-long reads, and Hi-C data. All of the genome sequences were assembled onto 24 chromosomes, and 48 telomeres were identified on both ends of all chromosomes. 99.29% complete BUSCOs were identified, demonstrating a high level of completeness. The average mapping ratio of short reads, PacBio HiFi reads, and ONT ultra-long reads aligned to the genome was more than 99.89%. 121.02 Mb repeating elements and 22,778 protein-coding genes were identified in the genome assembly. These results provide valuable resources for the evolutionary genomics research and the identification of key candidate genes for economic traits in marbled flounder.

## Background & Summary

Marbled flounder, *Pseudopleuronectes yokohamae* (FishBase ID: 1877), belonging to Pleuronectiformes, Pleuronectidae, is widely distributed in Northwest Pacific, from southern Hokkaidoof Japan to the Yellow Sea, the Gulf of Bo Hai, and the northern part of the East China Sea, including Korea^1–4^. It was once an important fishery resource, but due to overfishing, environmental pollution, and habitat degradation, its annual catch has decreased substantially from more than 500 t in 1980s to around 50 t in the 2000s^5–7^. In recent years, it has become an economically important aquaculture species in South Korea, Japan and north of China, because of its tolerance to low temperature, strong adaptability, delicate taste, and high nutritional value^8,9^.

Extensive studies of marbled flounder have been conducted, including life history and ecology^4,10^, nutrition^8^, growth^5^, reproductive biology^4,11^, genetic diversity and population structure^9,12–14^, sperm cryopreservation^15^, and stress response^16,17^. However, the lack of high-quality reference genome has greatly hindered the comprehensive genetic and functional genomic research. To date, only a contig-level marbled flounder genome with low continuity and completeness (contig N50 length of 2 kb assembled by Illumina sequencing) was reported^18^. Therefore, there is an urgent need to obtain a high-quality marbled flounder genome.

The rapid development of sequencing technologies and assembly algorithms, especially long-read sequencing technologies like PacBio high-fidelity (HiFi), and Oxford Nanopore Technologies (ONT) ultra-long sequencing, has made it possible to obtain high-quality telomere-to-telomere (T2T) genome. Several near-T2T or T2T genome assemblies have been reported in fish, such as Chinese sea bass (*Lateolabrax maculatus*)^19^, the giant grouper (*Epinephelus lanceolatus*)^20^, the tomato hind (*Cephalopholis sonnerati*)^21^, African catfish (*Clarias gariepinus*)^22^, Yadong trout (*Salmo trutta*)^23^, Asian icefish (*Protosalanx chinensis*)^24^, and zig-zag eel (*Mastacembelus armatus*)^25^.

**Fig. 1:**
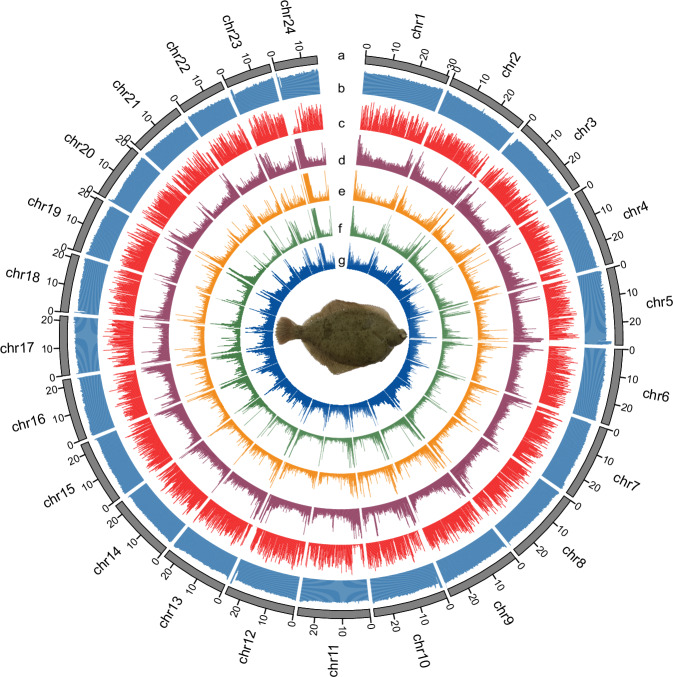
Features of marbled flounder T2T genome assembly. Tracks from outer to inner represent the chromosomes (**a**), GC content (**b**), distribution of gene density (**c**), distribution of the total repetitive sequences density (**d**), distribution of LTRs (**e**), distribution of LINEs (**f**), distribution of DNE TEs (**g**).

In the present study, we have assembled a gap-free T2T genome of marbled flounder combining whole genome short-read sequencing, PacBio HiFi sequencing, ONT ultralong sequencing, and Hi-C sequencing data. To our knowledge, this is the first gap-free T2T genome reported in flatfish (Fig. 1). This gap-free T2T genome assembly not only provides a robust foundation for identifying the key candidate genes of economic traits, but also advances the future evolutionary genomics within the family Pleuronectidae.

## Methods

### Sample collection, library construction and sequencing

A two-year-old female marbled flounder was collected for sampling from Zongzhe Marine Technology Co., Ltd, Yantai, Shandong, China. Fresh muscle tissues were sampled and stored at −80 °C for Genomic DNA (gDNA) extraction. Ten different tissues, including muscle, brain, pituitary, gill, liver, spleen, kidney, intestine, skin, and gonad, were collected for RNA extraction. For whole genome short-read sequencing and Hi-C sequencing, the gDNA was extracted using the TIANamp Marine Animals DNA Kit (Tiangen, China). Furthermore, for ONT ultra-long sequencing and PacBio HiFi sequencing, a standard sodium dodecyl sulfate (SDS) extraction method was used for the gDNA isolation. For RNA-seq sequencing, RNA was extracted using RNA Easy Fast Tissue/Cell Kit (Tiangen, China). The quality and quantification of gDNA and RNA was determined using agarose gel electrophoresis, NanoDrop One spectrophotometer (Thermo Fisher Scientific, USA), and Qubit 3.0 Fluorometer (Life Technologies, USA) to meet the requirements for library construction.

For short-read sequencing, a 200–400 bp short-read library was constructed using MGIEasy Universal DNA Library Prep Kit V1.0 (MGI, China) following the standard protocol, and the qualified library was sequenced on the DNBSEQ-T7RS platform (MGI, China). As a result, a total of 73.96 Gb (128×) of short-read sequencing data was generated (Table 1). For PacBio HiFi sequencing, a SMRTbell library was prepared using the SMRTbell Prep Kit 3.0 (Pacific Biosciences, CA, USA) according to the manufacturer’s instruction, and the qualified library then was performed on the PacBio Revio platform (Pacific Biosciences, CA, USA) for sequencing with the CCS mode. Finally, we obtained 31.47 Gb (54×) of PacBio HiFi sequencing data with an average and N50 read length of 17.41 kb and 17.51 kb, respectively (Table 1). The Hi-C library construction included cross-link with formaldehyde, DNA digestion, end repair and biotin labelling, blunt-end ligation, and DNA purification and shearing. After quality control, the library was implemented on DNBSEQ-T7RS platform for sequencing. In total, 58.98 Gb (102×) of Hi-C sequencing data was obtained (Table 1). For ONT ultra-long sequencing, the library was produced using the Oxford Nanopore SQK-ULK001 ligation kit, and the purified library was subsequently sequenced on a PromethION sequencer (Oxford Nanopore Technologies, Oxford, UK). Finally, 25.77 Gb (44 × ) of ONT ultralong sequencing data with mean and N50 read length of 101.84 kb and 100.00 kb, respectively (Table 1). To assist in annotating the coding genes, RNA from ten different tissues was mixed into a total RNA for RNA sequencing. A pair-ended sequencing library was constructed and sequenced on DNBSEQ-T7RS platform. A total of 16.53 Gb RNA-seq data were generated (Table 1).

**Table 1:** Statistics of the sequencing data.

Library Type	Sequencing Platform	Total Bases (Gb)	Reads Mean Length (bp)	Reads N50 (bp)	Q30 rate (%)
WGS short reads	DNBSEQ-T7	73.96	150	—	97.92
Hi-C	DNBSEQ-T7	58.98	150	—	92.76
Pacbio	Pacbio Revio	31.47	17,407	17,505	—
ONT	PromethION	25.77	101,848	100,000	—
RNA-seq	DNBSEQ-T7	16.53	150	—	97.57

### Genome assembly and telomere identification

**Fig. 2:**
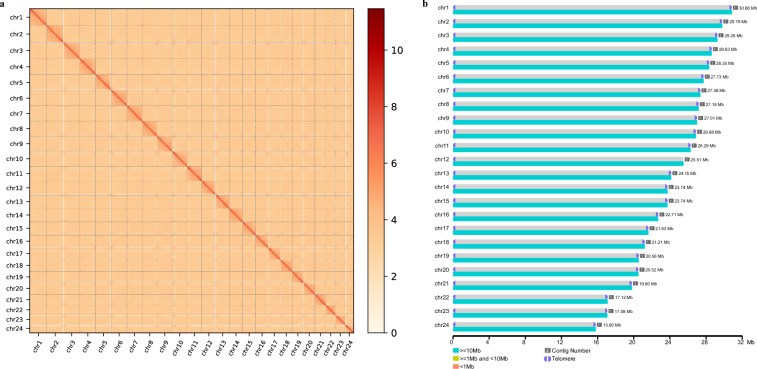
Hi-C heatmap and T2T genome display map of marbled flounder. (**a**) The Hi-C heatmap of chromosome interactions in marbled flounder. (**b**) Distribution map of telomeres and centromeres on the T2T marbled flounder genome. Purple semicircles indicate predicted telomeres.

We first evaluated the genome heterozygosity (0.71%) of marbled flounder using K-mer analysis. The primary contigs of marbled flounder genome were initially assembled using PacBio HiFi reads, ONT reads, and Hi-C data by Hifiasm (v0.19.9)^26^ with the parameter of “--n-hap 2”. The Purge Haplotigs pipeline (v1.1.3)^27^ with the parameter “-a 70--depth 250” was used to identify and remove haplotypic duplication of the primary genome. To obtain the chromosome-level genome, Hi-C reads were aligned to the contigs using BWA (v0.7.12)^28^ and filtered out low quality reads using a HiC-Pro pipeline (v3.1.0)^29^. After de-duplication, the valid pairs were used to anchor, order, and orient scaffolds onto chromosomes by juicer (v1.6)^30^ and 3D-DNA pipeline (v180992)^31^. Subsequently, the Hi-C contact map was interactively visualized and manually corrected using Juicebox (v1.91)^32^ based on the fact that the interaction within chromosomes is greater than that between chromosomes, the interaction at close range is greater than that at long range (Fig. 2a). Then, all ONT ultra-long reads were mapped to the chromosome-level genome using minimap2^33^ to extend the telomere sequences and were utilized to fill gaps by TGS-GapCloser^34^ to lengthen the contigs. After telomere extension and gap filling, Pilon (v1.23)^35^ was used to polish the genome assembly with short-read data. Finally, we obtained a gap-free T2T marbled flounder genome assembly with length of 582.73 Mb (contig N50 length of 26.29 Mb), which significantly improved the completeness and continuity compared to the published contig-level marbled flounder genome (GCA_000787555.1)^18^ (contig N50 length of 0.002 Mb) (Table 2). We further identified the occurrences of characteristic sequences (AACCCT/AGGGTT) in the telomere region by searching the whole genome using quartet (v1.2.5)^36^. As a result, a total of 48 telomeres were screened on both ends of 24 chromosomes (Fig. 2b and Table 3).

**Table 2:** Statistics of marbled flounder genome assemblies.

	GCA_051314325.1	GCA_000787555.1^18^
Total genome length (Mb)	582.73	547.80
Total chromosome length (Mb)	582.73	—
Number of chromosome	24	—
Number of contigs	24	525,502
Number of scaffolds	24	—
Contig N50 (Mb)	26.29	0.002
Scaffold N50 (Mb)	26.29	—

**Table 3:** Statistics of marbled flounder chromosomes and telomeres.

Chromosome	Length(bp)	Number of Telomeres	Number of gaps
chr1	30,859,922	1	0
chr2	29,635,121	1	0
chr3	29,259,407	1	0
chr4	28,634,317	1	0
chr5	28,352,015	1	0
chr6	27,732,592	1	0
chr7	27,379,010	1	0
chr8	27,124,858	1	0
chr9	27,007,596	1	0
chr10	26,883,915	1	0
chr11	26,290,307	1	0
chr12	25,510,882	1	0
chr13	24,151,384	1	0
chr14	23,743,414	1	0
chr15	23,742,429	1	0
chr16	22,713,765	1	0
chr17	21,626,437	1	0
chr18	21,213,813	1	0
chr19	20,557,026	1	0
chr20	20,517,144	1	0
chr21	19,800,744	1	0
chr22	17,115,885	1	0
chr23	17,079,772	1	0
chr24	15,795,157	1	0

### Repeat annotation

Repetitive sequences were predicted by a strategy of combining *de novo* prediction and homologous alignments. *de novo* repeat elements were predicted using RepeatMasker (v4.0.5)^37^ based on the *de novo* database generated by RepeatModeler (v1.0.8)^38^ and LTR-FINDER (v1.0.6)^39^. Homologue prediction was performed using RepeatMasker (v4.0.5) and RepeatProteinMask (v4.0.5) based on the Repbase library (v21.01)^40^. Furthermore, Tandem Repeats Finder (v4.0.7)^41^ was used to detect the tandem repeats. Finally, a total of 121.02 Mb of non-redundant repetitive sequences representing approximately 20.77% of the marbled flounder genome were predicted (Fig. 1 and Table 4). Among these repeats, DNA transposons (9.49%) were the most abundant, followed by long interspersed elements (LINEs, 3.42%), long terminal repeats (LTRs, 3.21%), and short interspersed nuclear elements (SINEs, 0.65%) (Fig. 1 and Table 4).

**Table 4:** Statistics of repetitive sequences in marbled flounder.

Type	Repbase TEs		TE protiens		*De novo*		Combined TEs	
	Length (bp)	% in genome	Length (bp)	% in genome	Length (bp)	% in genome	Length (bp)	% in genome
DNA	34,032,084	5.84	3,906,782	0.67	31,573,748	5.42	55,324,428	9.49
LINE	13,059,971	2.24	6,635,836	1.14	11,490,591	1.97	19,928,938	3.42
SINE	2,523,779	0.43	0	0.00	1,875,205	0.32	3,801,300	0.65
LTR	10,345,205	1.77	3,310,206	0.57	9,552,529	1.64	18,701,028	3.21
Satellite	3,122,222	0.54	0	0.00	3,276,266	0.56	6,312,932	1.08
Simple_repeat	0	0.00	0	0.00	0	0.00	0	0.00
Other	2,117	0.00	0	0.00	0	0.00	2,117	0.00
Unknown	613,113	0.11	10,068	0.00	26,311,504	4.52	26,745,069	4.59
Total	56,542,486	9.70	13,853,832	2.38	82,560,658	14.17	121,017,057	20.77

### Protein-coding gene prediction and functional annotation

**Fig. 3:**
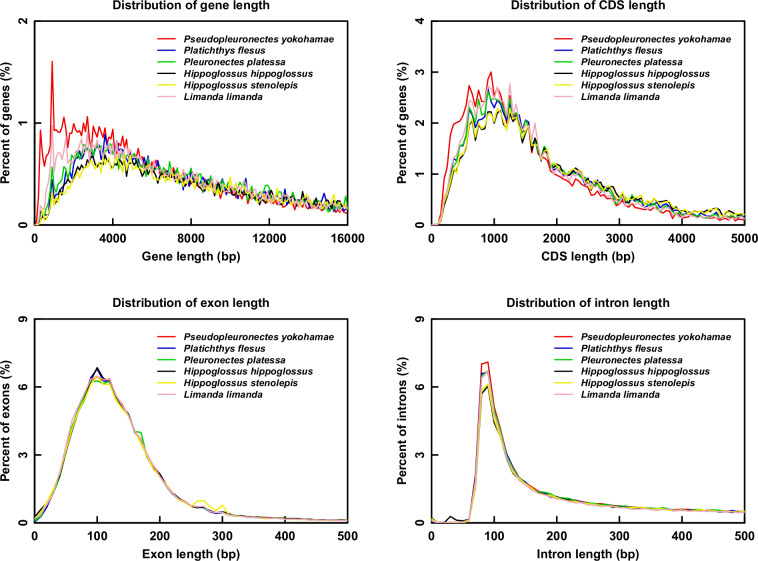
Comparisons of the statistical characteristics of the gene set elements between marbled flounder, *P. flesus*, *P. platessa*, *H. hippoglossus*, *L. limanda*, and *H. stenolepis*. (**a**) Gene length distributions. (**b**) CDS length distributions. (**c**) Exon length distributions. (**d**) Intron length distributions.

Gene structure prediction was performed through a combination of *de novo* prediction, homology-based prediction, and transcriptome-based prediction. For *de novo* prediction of the gene structures, GENSCAN (v1.0)^42^ and AUGUSTUS (v3.3.2)^43^ were used with default settings. For homology-based prediction, the protein sequences from 5 closely related representative fish species, including *Platichthys flesus*, *Pleuronectes platessa*, *Hippoglossus hippoglossus*, *Limanda limanda*, and *H. stenolepis*, were downloaded from NCBI and were then aligned to the marbled flounder T2T genome assembly to predict gene structure by miniprot(v0.11-r234)^44^ and Liftoff (v1.6.3)^45^ based on the homologous evidences. Moreover, RNA-seq data (accession number: SRR33264204) from ten mixed tissues were aligned to the starry flounder T2T genome using Hisat2 (v2.1.0)^46^ for transcriptome-based prediction. The mapped reads were assembled into transcripts using StringTie (v2.1.4)^47^. Subsequently, the merged transcripts were subjected to TransDecoder (v5.7.0, https://github.com/TransDecoder/TransDecoder) for protein-coding gene prediction. Finally, the above gene prediction results were integrated by MAKER2 (2.31.10)^48^, producing a non-redundant protein-coding gene set composed of 22,778 genes (Table 5). Comparisons of the statistical characteristics of the gene set elements between marbled flounder and other six related species exhibited a similar distribution patterns (Fig. 3).

**Table 5:** Statistics of predicted protein-coding genes in marbled flounder.

	Gene set	Number	Average gene length (bp)	Average CDS length (bp)	Average exon per gene	Average exon length (bp)	Average intron length (bp)
*De novo*	Genscan	29,396	13,873	1,616	9.09	177.74	1,515
	AUGUSTUS	30,204	8,873	1,313	7.33	179.17	1,195
Homolog	*Hippoglossus hippoglossus*	50,947	18,947	2,239	13.10	170.92	1,381
	*Platichthys flesus*	42,022	14,282	1,908	11.00	173.52	1,238
	*Limanda limanda*	34,961	13,246	1,784	9.91	179.88	1,286
	*Hippoglossus stenolepis*	45,454	18,530	2,251	13.08	172.12	1,348
	*Pleuronectes platessa*	42,610	13,994	1,927	10.59	181.87	1,258
Liftoff	*Platichthys flesus*	21,814	12,650	1,817	10.51	172.89	1,139
	*Pleuronectes platessa*	22,098	12,238	1,797	10.38	173.17	1,114
Trans ORF	RNAseq	12,671	15,241	1,883	12.09	291.07	1,057
MAKER		22,778	14,042	1,704	10.53	267.22	1,178

Functional annotation of protein-coding genes were performed by matching them with mutiple public protein databases, such as GO^49^, KEGG^50^, Pfam^51^, UniProt^52^, NR^53^, and InterPro^54^, using DIAMOND (v2.1.8)^55^ (E-value = 1e−5) or the corresponding inbuilt software. As a result, 22,386 genes, accounting for 98.28% of the predicted genes in marbled flounder, were annotated in at least one functional database (Table 6). In addition, the non-coding RNAs, including 2,703 tRNAs and 860 rRNAs, were annotated using tRNAscan-SE (v2.0.12)^56^ and BLASTN, respectively. 1,278 miRNAs and 1,318 snRNAs were annotated using Infernal (v1.1.4)^57^ based on Rfam database (Table 7).

**Table 6:** Statistics of functional annotation of protein-coding genes in marbled flounder.

		Number	Percent (%)
Total		22,778	
Annotated		22,386	98.28
	NR	22,996	98.14
	SwissProt	20,426	87.16
	TrEMBL	22,723	96.97
	KOG	18,826	80.34
	TF	5,585	23.83
	InterPro	21,922	93.55
	GO	16,755	71.50
	KEGG_ALL	22,635	96.59
	KEGG_KO	16,354	69.79
	Pfam	20,732	88.47
Unannotated		392	1.72

**Table 7:** Statistics of non-coding RNA in marbled flounder.

Type		Copy	Average length(bp)	Total length(bp)	% of genome
miRNA		1,278	87	110,867	0.019026
tRNA		2,703	75	202,733	0.034790
rRNA	rRNA	860	121	104,267	0.017893
	18S	2	1,123	2,246	0.000385
	28S	0	0	0	0
	5.8S	1	154	154	0.000026
	5S	857	119	101,867	0.017475
snRNA	snRNA	1,318	147	193,404	0.033177
	CD-box	189	126	23,903	0.0041
	HACA-box	77	153	11,755	0.002016
	splicing	1,042	149	155,764	0.02672
	scaRNA	10	198	1,982	0.00034

### Gene family and phylogenetic analysis

**Fig. 4:**
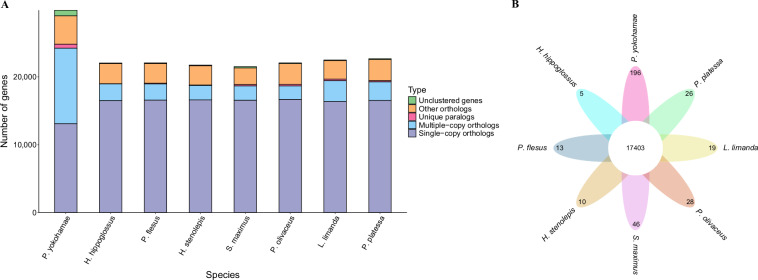
Comparison of gene families between marbled flounder and *H. hippoglossus*, *P. flesus*, *H. stenolepis*, *L. limanda*, *P. platessa*, *S. maximus*, and *P. olivaceus*. (**A**) Statistics of homologous gene numbers across 8 species. (**B**) Gene family clustering across 8 species.

**Fig. 5:**
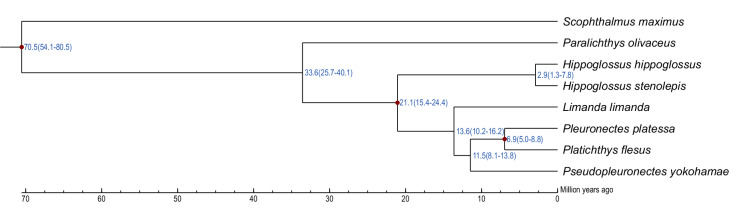
Phylogenetic relationships and divergence time estimation for marbled flounder and 7 other species in the Pleuronectidae family. *S. maximus* was chosen as an outgroup. All nodes were supported by 100 cycles of bootstrap resampling. Blue numbers represent the estimated divergence times with a 95% confidence interval. Divergences used for the recalibration of time estimation are marked by red dots.

Gene families of marbled flounder and the other seven species in the Pleuronectidae family, including *H. hippoglossus*, *P. flesus*, *H. stenolepis*, *L. limanda*, *P. platessa*, *Scophthalmus maximus*, and *Paralichthys olivaceus*, were identified and clustered by Orthofinder (v2.5.5)^58^. As a result, a total of 17,403 common families were obtained, which include 196 species-specific gene families (590 genes) identified in marbled flounder (Fig. 4B,C). Subsequently, 12,370 single copy genes were used for multiple sequence alignment using MAFFT (v7.525)^59^. Then, a maximum likelihood (ML) phylogenetic tree was constructed using RAxML (v8.2.12)^60^ and performed 1000 bootstrap replications. Additionally, MCMCtree program implemented in PAML (v4.9)^61^ was conducted to estimate divergence times among species. The result showed that marbled flounder began to diverge around 11.5 million years ago (Fig. 5).

## Data Records

The WGS short-read sequencing data have been uploaded to NCBI Sequence Read Archive (SRA) database with the accession number SRR33323076^62^.The PacBio HiFi sequencing data have been deposited at NCBI SRA database with the accession number SRR33323077^63^. The Hi-C sequencing data have been submitted to NCBI SRA database with the accession number SRR33323078^64^. The ONT sequencing data have been deposited into NCBI SRA database with the accession number SRR33323079^65^. RNA-seq data can be obtained under the accession number SRR33264204^66^. The genome assembly has been submitted to the NCBI Genbank with accession number GCA_051314325.1^67^. The genome annotation file, gene CDS, and protein data have been deposited at Figshare^68^.

## Technical Validation

Compared to the previously published marbled flounder genome, the contig N50 length of our T2T genome assembly increased from 2.00 kb to 26.29 Mb, exhibiting a substantial enhancement in contiguity of the genome assembly. The completeness of the marbled flounder genome assembly was assessed using BUSCO (v.5.1.2) with the actinopterygii_odb10 database containing 3,640 BUSCOs. The results showed that 3,614 (99.29%) complete BUSCOs were identified, which is higher than that of closely related species, such as *Platichthys flesus* (98.2%)^69^ and *Pleuronectes platessa* (97.9%)^70^, indicating a high level of integrity. Moreover, the base-level accuracy and the consensus quality value (QV) was evaluated using Merqury^71^, and the average per-base accuracy rates and QV were 0.99986 and 38.52, respectively, highlighting a high-quality assembly. Furthermore, WGS short reads, PacBio HiFi reads, and ONT ultra-long reads were aligned to the genome assembly using bwa (v0.7.17)^28^ and minimap2 (v2.17)^33^. The average mapping rates and coverage were more than 99.89% and 99.70%, respectively, highlighting a high sequences consistency.

## Data Availability

The genome sequencing data, including WGS short-read, PacBio HiFi, Hi-C, and ONT ultra-long, can be publicly available from the NCBI SRA database with the accession number SRR33323076^62^, SRR33323077^63^, SRR33323078^64^, and SRR33323079^65^. RNA-seq data used for genome annotation also can be publicly obtained from the NCBI SRA database with the accession number SRR33264204^66^. The genome assembly can be publicly available from the NCBI Genbank with accession number GCA_051314325.1^67^. The genome annotation file, gene CDS, and protein data can be publicly downloaded at Figshare^68^ (10.6084/m9.figshare.28823984.v2).

## Code availability

All software and tools were used in this study following the instructions and protocols provided by the respective software developers. The software versions and corresponding parameters applied have been described in the Methods section, and default parameters were used if no parameter was described. No custom code was used in this work.
